# Patient and work flow and costs associated with staff time and facility usage at a comprehensive cancer centre in Quebec, Canada – a time and motion study

**DOI:** 10.1186/1472-6963-12-370

**Published:** 2012-10-29

**Authors:** Gayle A Shinder, Pierre Emmanuel Paradis, Marianne Posman, Natalia Mishagina, Marie-Pascale Guay, Dina Linardos, Gerald Batist

**Affiliations:** 1 Department of Oncology, McGill University, Gerald Bronfman Centre, 546 Avenue des Pins Ouest, Montreal, Quebec, H2W 1S6, Canada; 2 Groupe d'analyse ltée, 1000 de la Gauchetière West, Suite 1200, Montreal, Quebec, H3B 4W5, Canada; 3 Current address: AppEco Analytics, 222 Louis-Ducharme, Mont-Saint-Hilaire, Quebec, J3H 6J6, Canada; 4 Segal Cancer Centre of the Lady Davis Institute for Medical Research, Jewish General Hospital, 3755 Côte Ste-Catherine Road, Montreal, Quebec, H3T 1E2, Canada; 5 Current address: CIRANO, 2020 University Street, Room 2520-B, Montreal, Quebec, H3A 2A5, Canada

**Keywords:** Patient flow, Work flow, Time and motion, Cost analysis, Metastatic colorectal cancer, FOLFOX/bevacizumab, XELOX/bevacizumab

## Abstract

**Background:**

Mapping patient and work flow and cost analysis studies can help determine the most efficient and cost effective way of providing health services while still maintaining the best standards of care. This study used both time and motion methodology and hospital data to assess the contribution of staff time and facility usage to the overall cost of cancer care during patient visits to a comprehensive cancer centre in Quebec, using metastatic colorectal cancer as a model.

**Methods:**

A workflow diagram was created mapping direct and indirect steps involved during a patient’s physician or treatment (FOLFOX/bevacizumab or XELOX/bevacizumab) visit. Staff were timed as they performed each task and this data together with compensation amounts were used to calculate personnel costs. Mean work times and 95% confidence intervals (CI) were calculated. Operation and maintenance (O&M) costs for the Centre were calculated using information from hospital databases. All costs were presented in constant Canadian dollars for the 2010–2011 fiscal year period.

**Results:**

For physician visits, direct and indirect personnel costs were $9.25 (95%CI:$7.00-$11.51) and O&M costs were $60.21, for a total of $69.46 (95%CI:$67.21-$71.72). For treatment visits, personnel and O&M costs were $71.91 (95%CI:$45.53-$98.29) and $62.00 respectively for a total of $133.91 (95%CI:$107.53-$160.29). When calculated for treatment alone, the total cost was $136.06 (95%CI:$109.16-$162.95) for FOLFOX/bevacizumab and $119.94 (95%CI:$96.89-$142.99) for XELOX/bevacizumab. The highest cumulative personnel costs were for the pharmacists and nurses ($38.87 and $34.82 respectively). Regarding patient flow, total time in between steps was 77.6 and 49.5 minutes for a physician or treatment visit respectively.

**Conclusions:**

This study from a health care provider’s perspective, demonstrated that in the context of increasingly expensive therapies, costs associated with staff time and facility usage do not contribute greatly to the overall cost of treating cancer at this cancer centre. It also illustrated the need for improvements in patient and work flow to reduce wait times in the clinic.

## Background

With the increased use of health care services due to the aging population as well as population growth, and the ballooning health care costs, it has become apparent that changes need to be made to increase efficiency and reduce costs while maintaining optimal standards of care. In Canada, the number of new cancer cases is expected to increase 75% in 25 years from 160,000 in 2006 to 280,000 in 2031 [1]. It has been estimated that the annual cost for providing treatment and services to cancer patients in Canada is approximately $5 billion (1). These challenges are not unique to Canada. Institutions around the world are performing cost analysis studies to determine the breakdown of costs for treating particular cancers, or to compare the cost effectiveness of different treatment regimens, in order to assess the financial burden on their health care system [2-8].

Time and motion studies have long been used to assess work processes. In health care delivery, such studies are performed, notably to modify existing procedures, implement new ones, improve efficiency and reduce costs. In the early 1990’s, a time and motion study was conducted to evaluate the efficiency of multileaf collimators (MLC) in the delivery of radiotherapy [9]. Another study which compared three types of surgical sessions in a dermatology clinic identified not only the most efficient method, but also which work steps were causing delays in the provision of service [10]. A time and motion study was also implemented to look at the time spent on various tasks by medical-surgical nurses to identify ways of improving nursing care [11]. Time and motion studies can also be used to measure the overall cost of performing routine screening procedures such as a colonoscopy [12] as well as the utilization of resources and time involved in treating co morbidities of chronic diseases such as cancer [13]. In the oncology clinic this methodology has been used to determine the time and cost of administering certain treatments to cancer patients [14], and to compare the overall cost of two different cancer treatments [15]. In a hematology-oncology inpatient unit a time and motion study was implemented to assess daily workload of nurses [16].

The Segal Cancer Centre located at the Jewish General Hospital in Montreal was opened in 2006 to meet the growing need for a state of the art comprehensive cancer facility within the hospital to replace the previous small and over-crowded oncology clinic. The hospital is affiliated with McGill University and services patients from across the province of Quebec. In accordance with Canadian trends, the Centre has seen a dramatic increase in the number of patients resulting in increased costs to the health care system and space constraints during busy clinic times.

The purpose of this study was to assess from a health care provider’s perspective, the contribution of staff time and facility usage to the cost of treating patients at the Segal Cancer Centre using metastatic colorectal cancer as a model, with the treatment regimens being either FOLFOX (Folinic Acid, Fluorouracil, Oxaliplatin)/bevacizumab or XELOX (Capecitabine, Oxaliplatin)/bevacizumab. The question is whether these costs add considerably to drug costs, or are the cost of the drugs themselves the most significant challenge to the health care system. A secondary goal was to map patient and work flow during a patient’s physician and treatment visit to assess efficiency. For this purpose, a ‘real-time’ analysis based on time and motion methodology was performed.

Colorectal cancer is ideal as a model for this study because it is the third most common cancer in Canada, affecting both men and women [17]. In the past, median survival for metastatic colorectal cancer was measured in months, but more recently we and others have reported extension of life beyond three years [18,19]. Since as many as 40% of all colorectal cancer patients will at some point metastasize, this represents an important point in the disease trajectory from a health resource viewpoint. Furthermore, because survival with metastatic colorectal cancer is progressively improving, including a small number of patients who might have very long term disease-free survival as a result of surgical resection of metastases, this represents an increasingly important patient group.

While cost analysis studies have been done to determine the medical costs of treating cancer from a health care provider’s or payer’s perspective, to our knowledge, this is the first study that uses a combination of time and motion methodology for mapping patient and work flow and assessing staff time, and hospital data for determining facility usage, to ascertain their specific contributions to the overall costs of treating cancer.

## Methods

### Time and motion study

**Figure 1:**
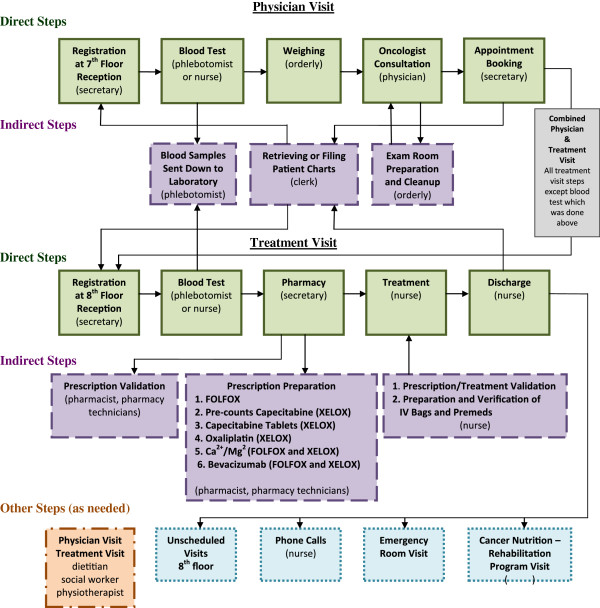
Workflow Diagram – Physician and Treatment Visits.

Following discussions with a number of health care providers and administrative staff, a workflow diagram was created to map the direct and indirect work steps and type of personnel involved during physician or treatment visits (Figure 1). Signed consent forms were obtained from all staff and patients who participated in the study. A research assistant (RA) was hired to serve as the timekeeper. Eligible patients were those with metastatic colorectal cancer age 18 years or older undergoing treatment with FOLFOX/bevacizumab or XELOX/bevacizumab. The RA followed the patient from step to step during the duration of the visit and used a stopwatch to record the time required for the employee to perform the task(s). For patient privacy reasons, at the oncologist consultation step timing began from the moment the patient entered the examination room, even if the physician did not immediately enter.

Figure 1 shows the nine steps where staff directly interacted with the patient during the physician or treatment visit (green boxes). On occasion a patient would speak to a dietitian, social worker or physiotherapist during either visit (Other Steps, orange box) and this interaction was timed if the RA was present. During the treatment step, at various times the nurse administered the different drugs involved in the treatment regimen (premedication, 5-fluorouracil, leucovorin, oxaliplatin, bevacizumab, calcium/magnesium), talked to the patient or checked the drug infusion pump. To afford the patient and family as much privacy as possible, the RA did not remain with the patient during the entire course of treatment. Instead, she timed the work steps involved in starting the treatment, then returned to time each medication changeover, and then the discharge step. The duration of time the nurse spent with the patient during the treatment step was determined by adding up the individual times. The discharge time was calculated as the length of time required to disconnect the pump and measure vital signs, as well as any other interactions with the nurse or other staff prior to the patient leaving the premises. Direct steps that took under 1 minute to perform were rounded up to 1 minute. For steps taking longer than 1 minute, anything equal to or above the 10 second mark was rounded up to the nearest minute and anything below was rounded down.

There were twelve indirect steps (Figure 1, lavender boxes) performed in preparation for, or as a result of, the direct steps, and four possible post-treatment steps (Figure 1, Other Steps, blue boxes) as required. The times for the post-treatment steps were mainly determined by chart review or from the staff person involved.

Since this study did not involve more than minimal risk (in accordance with article 1.6 of the Canadian Tri-Council Policy Statement of Ethical Conduct for Research Involving Humans), the Jewish General Hospital’s Research Ethics Office granted expedited approval as protocol #09-129, with corroborative approval of the Research Ethics Committee.

### Cost analysis

For the time and motion portion, four statistics are reported: the number of timed observations, the mean duration of each timed step, and the 95% confidence intervals (CI) around the mean duration, calculated using a two-sided t-Student distribution. Time results are reported in minutes both for the visit duration and total worked time (i.e. all minutes worked by all employees, or person-minutes) during each visit step. The cost analysis component of the study involved the collection of information about the Centre’s costs for the fiscal year April 1, 2010-March 31, 2011, from the hospital databases. All costs are presented in constant Canadian dollars during this 2010–2011 time period. The unit cost of a visit consists of personnel costs and operation and maintenance (O&M) costs. Personnel costs represent the cost of work performed by Segal Cancer Centre staff during the direct and indirect work steps and were obtained by multiplying the mean duration of each work step by the total hourly rate (including benefits) of the specific personnel category involved in the task. O&M costs consist of equipment (in the exam rooms, treatment area, pharmacy and laboratory) and other operational costs (sterilization, laundry/dry cleaning, biomedical waste management, operational facilities, security, maintenance and repair, mail, telecommunications). Equipment costs were calculated based on the estimated acquisition costs in 2010–2011 of existing Segal Cancer Centre equipment (exam tables, diagnostic kits, digital thermometers, recliners, treatment beds, IV pumps, crash cart, automatic NIBP, computerized narcotic dispensing machine, digital scale, nurse call system, pharmacy hoods, refrigerators and freezers), their expected useful life span, and the number of equipment items purchased each year. Other O&M costs were drawn from the overall hospital costs, and attributed to the Segal Cancer Centre based on (a) the share of patient visits to the Segal Cancer Centre (sterilization, laundry/dry cleaning, biomedical waste management) or (b) the floor area of the Segal Cancer Centre relative to the entire hospital (operational facilities, security, maintenance and repair, mail, telecommunications). Total O&M costs per visit were obtained by dividing total costs attributable to the Segal Cancer Centre by the total number of patient visits at the Centre.

Since physician salaries and pharmaceutical costs are covered by Quebec’s public health care plan (“Régie de l’assurance-maladie du Québec”) rather than by budgets of the Segal Cancer Centre or of the Jewish General Hospital, these costs are classified as “medical” and, as such, were excluded from our cost calculations. However, to provide an overall picture of economic costs, the Discussion section compares the costs of the contribution of staff time and facility usage, which are our main focus, with physician salaries and pharmaceutical costs.

All analyses were conducted using Microsoft® Office Excel 2003 (Microsoft Corporation, Redmond, WA). Statistical significance is defined as a two-sided α-level of 0.05 or less.

## Results

Twenty-four patients (14 female, 10 male) with a mean age of 63 (41–85) were followed. There were a total of 48 physician visits (36 physician only and 12 physician/treatment) and 60 treatment visits (48 treatment only and 12 physician/treatment). Of the treatment visits, 52 (86.7%) were FOLFOX/bevacizumab and 8 (13.3%) were XELOX/bevacizumab.

Table 1 shows the mean time of each direct work step and the mean time worked by the employee for both the physician and treatment visits. For some work steps there were fewer observations (n) due to missed timings for a variety of reasons. The average total duration of the physician visit was 129.2 (95%CI:114.2-144.2) minutes, whereas the mean duration of the timed work steps was 51.6 (95%CI:46.3-57.1) minutes. For treatment visits, the average total duration was 393.0 (95%CI:374.9–411.1) minutes, with timed work steps taking up 343.5 (95%CI:328.1–358.7) minutes. Therefore, waiting or in-transit times were 77.6 minutes during physician visits (60% of visit time), and 49.5 minutes during treatment visits (13% of visit time).

**Table 1:** Direct Work Steps: Average duration of each step and time worked by employee

**Direct Work Steps**	**n**	**Mean Time (min)**
		**(95% Confidence Interval)**
**Physician Visits**		
***Average Total Duration of Physician Visit***	***48***	***129.2 (114.2 – 144.2)***
7^th^ floor registration (secretary) ^a, b^	45	1.3 (1.1 - 1.6)
Blood Test – Physician Visit (phlebotomist or nurse) ^a,b^	36	3.6 (2.7 - 4.5)
1. phlebotomist*	27	2.7 (2.0 - 3.4)
2. nurse*	9	6.2 (3.4 - 9.0)
Weighing (orderly) ^a, b^	42	1.0 (0.9 - 1.1)
Appointment with Oncologist (physician) ^a^	48	33.3 (28.4 - 38.2)
Appointment Booking (secretary) ^a, b^	44	12.1 (10.1 - 14.1)
Other - Physician Visit (secretary, nurse, physiotherapist) ^a, b^	48	0.3 (−0.1 - 0.7)
*Total mean duration of timed visit steps*^a^		*51.6 (46.3 - 57.1)*
*Total mean time worked by employees (excluding physicians)*^b^		*18.3 (14.1 – 22.6)*
**Treatment Visits**		
***Average Total Duration of Treatment Visit***	***60***	***393.0 (374.9 – 411.1)***
8^th^ floor registration (secretary) ^c, d^	36	1.1 (1.0 - 1.2)
Blood Test – Treatment Visit (phlebotomist or nurse) ^c, d^	35	9.9 (6.8 - 13.0)
1. phlebotomist*	12	2.9 (2.1 - 3.7)
2. nurse*	23	13.5 (9.3 - 17.7)
Pharmacy (secretary) ^c, d^	47	1.4 (1.1 - 1.6)
Treatment (overall treatment time) ^c^	60	322.7 (308.1 - 337.4)
Treatment (nurse time) ^d^	60	24.6 (18.2 - 31.0)
Oncologist consultation during treatment visit ^c^	48	0.5 (0.0 - 0.9)
Discharge (overall discharge time) ^c^	58	6.3 (3.3 - 9.3)
Discharge (nurse time) ^d^	58	11.0 (−1.0 - 23.1)
Other- Treatment Visit (dietitian, social worker) ^c, d^	60	1.6 (0.5 - 2.8)
*Total mean duration of timed visit steps*^c^		*343.5 (328.1 - 358.7)*
*Total mean time worked by employees*^d^		*49.6 (26.1 – 73.1)*
**Other Visits** (determined by chart review)		
Unscheduled visits to clinic	10	121.8
Phone calls (nurse)	3	20.0
Cancer Nutrition-Rehabilitation Program	3	139.0
Emergency Room visits	11	794.8

^a^Applicable for duration of work step.

^b^Applicable for time worked by employee.

^c^Applicable for duration of work step.

^d^Applicable for time worked by employee.

*Whether blood is taken by the nurse or phlebotomist is dependent on the special circumstances of the patient, therefore when calculating the total mean time worked by employees the combined mean time was used rather than the individual mean times for the phlebotomist and the nurse.

Registration for physician or treatment visits, weighing and checking in at pharmacy were quick steps, i.e. 1–2 minutes. The mean time for blood drawing was 3.6 (95%CI:2.7–4.5) and 9.9 (95%CI:6.8–13.0) minutes for the physician and treatment visits respectively, and was either done by a nurse or a phlebotomist, depending on whether or not the patient had a portacath for vascular access. The appointment-booking time, 12.1 (95% CI:10.1–14.1) minutes, is fairly long due to the lengthy procedures required to contact other clinics and book scans and other procedures for the patient. The mean time for the consultation with the oncologist was 33.3 (95%CI:28.4–38.2) minutes. Thus for hospital staff only, the mean time worked per physician visit was 18.3 (95%CI:14.1-22.6) minutes.

The average nurse time with the patient during treatment was 24.6 (95%CI:18.2–31.0) minutes out of the total treatment duration of 322.7 (95%CI:308.1–337.4) minutes. Overall discharge time was 6.3 (95%CI:3.3–9.3) minutes, however the nurse time was 11.0 (95%CI:-1.0–23.1) minutes because in some cases there were complications during discharge which required the presence of more than one nurse. The average time worked by employees during the treatment visit was 49.6 (95%CI:26.1–73.1) minutes. While dietitians, social workers and physiotherapists are sometimes required by patients, in our particular study this was not a frequent occurrence.

Complications following treatment often result in unscheduled patient visits to the clinic, emergency room visits, phone calls to the nurse, and/or appointments at the Cancer Nutrition-Rehabilitation Program clinic. Since these steps were not timed by the RA and there was limited information, they were not included in the cost analysis.

Table 2 shows the mean time worked by employees for each of the indirect steps, which amounted to totals of 2.7 (95%CI:2.3–3.1) and 66.5 (95%CI:59.6–73.2) minutes for physician and treatment visits, respectively. For treatment visits, employee time was longer for patients treated with FOLFOX/bevacizumab, 70.0 (95%CI:67.1–72.7) minutes, than with XELOX/bevacizumab, 43.7 (95%CI:41.2–46.0) minutes. The actual preparation of FOLFOX took 39.6 (95%CI:35.9-43.3) minutes of employee time, whereas the preparation of XELOX took a total of 13.3 minutes (capecitabine pre-counts: 2.0 (95%CI:2.0-2.0) minutes; capecitabine preparation: 3.4 (95%CI:2.3-4.5) minutes, oxaliplatin preparation: 7.9 (95%CI:7.2-8.5) minutes).

**Table 2:** Indirect Work Steps: Average duration of each step and time worked by employee

**Indirect Work Steps**	**n**	**Mean Time (min)**
		**(95% Confidence Interval)**
**Physician Visits**		
Exam room preparation and cleanup (orderly)	27	1.1 (1.0 - 1.2)
Sending tubes of blood down to laboratory (phlebotomist)	10	1.0 (0.8 - 1.2)
Retrieving and filing patient charts (clerk)	300	0.6 (0.6 - 0.7)
**Total mean time worked by employees**		**2.7** (2.3 - 3.1)
**Treatment Visits**		
**FOLFOX (n=52); XELOX (n=8)**		
Sending tubes of blood down to laboratory (phlebotomist) ^a, b^	10	1.0 (0.8 - 1.2)
Retrieving and filing patient charts (clerk) ^a, b^	300	0.6 (0.6 - 0.7)
Prescription validation (pharmacist) ^a, b^	16	5.3 (4.0 - 6.5)
Preparation of calcium and magnesium ^a, b^	44	6.5 (6.0 - 6.8)
1. pharmacist	44	2.8 (2.8 - 2.8)
2. pharmacy technician	44	3.7 (3.3 - 4.0)
Preparation of FOLFOX (5-FU, leucovorin, oxaliplatin) ^a^	6	39.6 (35.9 - 43.3)
1. pharmacist	6	18.6 (14.5 - 22.7)
2. pharmacy technician	6	21.0 (16.7 - 25.2)
Pre-counts of capecitabine and labeling (pharmacy technician) ^b^	10	2.0 (2.0 - 2.0)
Preparation of capecitabine tablets for XELOX treatment ^b^	6	3.4 (2.3 - 4.5)
1. pharmacist	6	0.5 (0.2 - 0.8)
2. pharmacy technician	6	2.9 (2.1 - 3.7)
Preparation of oxaliplatin for XELOX treatment ^b^	9	7.9 (7.2 - 8.5)
1. pharmacist	9	3.9 (3.4 - 4.4)
2. pharmacy technician	9	4.0 (3.5 - 4.4)
Preparation of bevacizumab ^a, b^	6	10.1 (8.4 - 11.8)
1. pharmacist	6	4.9 (3.8 - 6.0)
2. pharmacy technician	6	5.2 (3.6 - 6.9)
Prescription/treatment verification (nurse) ^a, b^	14	4.5 (−0.8 - 9.8)
Preparation and verification of IV bags, premeds (nurse) ^a, b^	54	2.4 (1.9 - 3.0)
**Total mean time worked by employees** (all indirect steps) (Based on 86.7% FOLFOX/bevacizumab – n=52; 13.3% XELOX/bevacizumab – n=8)		**66.5** (59.6 - 73.2)
**Total mean time worked by employees**		
**1. FOLFOX/bevacizumab treatment**^**a**^		**70.0** (67.1 - 72.7)
**2. XELOX/bevacizumab treatment**^**b**^		**43.7** (41.2 - 46.0)

^a^Applicable to FOLFOX/bevacizumab treatment.

^b^Applicable to XELOX/bevacizumab treatment.

Table 3 shows the personnel (direct and indirect steps) and O&M costs per patient visit. For the physician visit, they were $9.25 (95%CI:$7.00-$11.51) and $60.21 respectively, for a total of $69.46 (95%CI:$67.21-$71.72). For the treatment visit they were $71.91 (95% CI:$45.53-$98.29) and $62.00 respectively, for a total of $133.91 (95%CI:$107.53-$160.29). The total costs per treatment visit for FOLFOX/bevacizumab and XELOX/bevacizumab were $136.06 (95%CI:$109.16-$162.95) and $119.94 (95%CI:$96.89-$142.99) respectively.

**Table 3:** Personnel and O&M costs per patient visit (C$)

	** Costs for 2010–2011**
	**(95% Confidence Interval)**
** Physician Visits**	
**Personnel Costs**	**$9.25** ($7.00 - $11.51)
1. Direct work steps	$8.08 ($6.00 - $10.16)
2. Indirect work steps	$1.17 ($1.00 - $1.35)
**Operation and Maintenance (O&M) Costs**	**$60.21 (NA)**
1. O&M cost excluding equipment	$22.44 (NA)
2. Specific equipment costs	$37.77 (NA)
**Total cost for physician visit**	**$69.46** ($67.21 - $71.72)
** Treatment Visits**	
**Personnel Costs**	**$71.91** ($45.53 – $98.29)
1. Direct work steps ^a, b^	$31.80 ($16.53 - $47.07)
2. Indirect work steps	$40.11 ($28.99 – $51.22)
3. FOLFOX/bevacizumab ^a^	$42.26 ($30.63 - $53.88)
4. XELOX/bevacizumab ^b^	$26.14 ($18.36 - $33.93)
**Operation and Maintenance Costs**^a, b^	**$62.00 (NA)**
1. O&M cost excluding equipment	$22.44 (NA)
2. Specific equipment costs	$39.56 (NA)
**Total cost for treatment visit**	**$133.91** ($107.53 – $160.29)
1. FOLFOX/bevacizumab^a^	$136.06 ($109.16 - $162.95)
2. XELOX/bevacizumab	$119.94 ($96.89 - $142.99)

^a^Applicable to FOLFOX/bevacizumab treatment.

^b^Applicable to XELOX/bevacizumab treatment.

Table 4 shows the breakdown of mean time worked by employee category and their respective personnel costs. For pharmacy, although the cumulative mean time worked was similar for the technician and pharmacist (47.7 and 48.8 minutes respectively), the personnel cost for the pharmacist was higher ($38.87 versus $18.95). The total pharmacy staff time for the preparation of FOLFOX/bevacizumab was longer than for XELOX/bevacizumab (61.4 versus 35.1 minutes) and is more expensive with respect to personnel costs ($36.97 versus $20.85). Regarding nursing, the treatment step was the most time consuming (24.6 minutes) and therefore the most costly ($16.18 out of a total cost of $34.82). For secretarial staff, appointment booking was the most time consuming (12.1 minutes) and costly ($4.86) step. The combined personnel costs for the phlebotomist, orderly, clerk and other were just over $5.00. Thus, of all the staff involved, it was pharmacist and nurse time which resulted in the highest personnel costs.

**Table 4:** Breakdown of mean time worked and costs by personnel (C$)

**Category**	**Mean Time Worked**	**Cost**	**Portion of Cost**	**Portion of Cost**
	**(min)**		**Physician Visit**	**Treatment Visit**
**1. Pharmacy**				
*a. Technician*				
i. Preparation of FOLFOX/bevacizumab	29.9	$11.88		$11.88
ii. Preparation of XELOX/bevacizumab	17.8	$7.07		$7.07
*b. Pharmacist*				
i. Preparation of FOLFOX/bevacizumab	31.5	$25.09		$25.09
ii. Preparation of XELOX/bevacizumab	17.3	$13.78		$13.78
**Total pharmacy for FOLFOX/**bevacizumab (n=52)	**61.4**	**$36.97**		**$36.97**
**Total pharmacy for XELOX/**bevacizumab (n=8)	**35.1**	**$20.85**		**$20.85**
**Treatment Average** (86.7% FOLFOX; 13.3% XELOX)		**$34.81**		**$34.81**
**2. Nursing**				
a. Blood (physician and treatment visits)*	10.4	$6.84	$1.02	$5.82
b. Treatment	24.6	$16.18		$16.18
c. Discharge	11.0	$7.25		$7.25
d. Prescription verification	4.5	$2.94		$2.94
e. Preparation of IV bags	2.4	$1.61		$1.61
**Total**	**52.9**	**$34.82**	**$1.02**	**$33.80**
**3. Phlebotomist**				
a. Blood (physician and treatment visits)*	3.0	$1.61	$1.08	$0.53
b. Sending tubes of blood down to laboratory (physician and treatment visits)	2.0	$1.04	$0.52	$0.52
**Total**	**5.0**	**$2.65**	**$1.60**	**$1.05**
**4. Secretary**				
a. 7^th^ floor registration	1.3	$0.53	$0.53	
b. Appointment booking	12.1	$4.86	$4.86	
c. 8^th^ floor registration	1.1	$0.43		$0.43
d. Pharmacy secretary	1.4	$0.55		$0.55
**Total**	**15.9**	**$6.37**	**$5.39**	**$0.98**
**5. Orderly**				
a. Weighing	1.0	$0.38	$0.38	
b. Exam room preparation	1.1	$0.43	$0.43	
**Total**	**2.1**	**$0.81**	**$0.81**	
**6. Clerk**				
Retrieving and filing patient charts (physician and treatment visits)	**1.2**	**$0.46**	**$0.23**	**$0.23**
**7. Other**				
(physician and treatment visits)	**2.0**	**$1.24**	**$0.20**	**$1.04**
**TOTAL**		**$81.16**	**$9.25**	**$71.91**

*Whether blood is taken by the nurse or phlebotomist is dependent on the special circumstances of the patient, therefore the mean time worked was calculated using n= combined number of observations for the nurse and for the phlebotomist.

## Discussion

Patients treated at the Segal Cancer Centre proceed through a standard route when they arrive for an appointment with their oncologist or for receipt of their treatment. In this study we mapped out that route, determined the length of time staff spend attending to the patient at each step and what that translates into with regards to personnel costs. We also assessed the cost of usage of the oncology clinic and treatment area which encompasses two floors of one pavilion in the hospital.

 Cost analysis studies from different countries comparing XELOX and FOLFOX have routinely shown the former to be less expensive when taking into account the cost of purchasing and administering the drugs, the management of serious adverse events and other medical resource utilization costs [5-8]. In contrast, our study focused on the contribution of staff time and facility usage to the cost per patient visit and demonstrated that it was only approximately 12% lower with XELOX. Thus it appears that the self-administered oral medication, capecitabine, has no significant financial advantage with regards to staff time and facility usage because of the preparation and administration of drugs requiring intravenous access and infusion (oxaliplatin and bevacizumab).

The 2011 drug costs of FOLFOX/bevacizumab (modified FOLFOX6, bevacizumab, calcium and magnesium) and XELOX/bevacizumab (capecitabine, bevacizumab, oxaliplatin, calcium and magnesium) were $3,324/cycle given every two weeks and $5,603/cycle given every three weeks, respectively. The physician fee per follow-up visit, which usually occurs every second treatment, was $41.90. Taking into account the personnel and O&M costs determined here for each treatment ($136.06/cycle and $119.94/cycle respectively), in a 12-week time span the cost per patient of administering the treatment is $20,886 for FOLFOX/bevacizumab and $22,976 for XELOX/bevacizumab.

One striking outcome of this analysis is the demonstration that for the treatment of metastatic colorectal cancer, personnel and O&M costs are minimal, with drug costs contributing >95% of the total cost. This finding holds true across the entire calculated confidence intervals, which reflect the sensitivity of time results. Indeed, because all costs in the model were based on mean values of collected data on actual time rather than deterministic fixed parameters, the uncertainty in the model is captured through confidence intervals rather than sensitivity analyses. Given that the patient flow and work steps are similar across cancer types at the Segal Cancer Centre, this trend may well be the same for other cancers with some minor variations depending on the cancer type and the cost of treatments.

There were several limitations to this study. Indirect steps were determined separately from the direct steps and therefore were not linked to a particular patient visit. Furthermore, some indirect steps were performed in batches (e.g. blood samples sent to laboratory, preparation of calcium/magnesium) rather than individually for each patient visit. Although every effort was made to time all direct work steps of each patient visit, due to unforeseen circumstances, for some visits the timing could not be completed for particular steps, (e.g. registration, blood taking). However, given the consistency of the timings that had been obtained, the missed ones would likely not have significantly altered the outcome. Our time and motion protocol did not include assessing the time spent for unscheduled visits to the Centre for management of serious adverse events and symptom control issues. Moreover, during the treatment step the RA was only present for medication changeovers, which may have resulted in an underestimation of these work times. However, it is not likely that additional costs associated with these limitations would have significantly increased the impact of the personnel costs as compared with the cost of the drugs.

This time and motion study also provided a detailed description of the patient trajectory through our cancer centre, giving a glimpse of the complexity of care of these patients. The inefficiencies in patient flow, particularly specific points with exaggerated wait times, correspond directly with dissatisfaction consistently identified in two patient satisfaction surveys conducted by the Jewish General Hospital’s Quality Program. Furthermore, simple observation of the use of the common space (corridors, waiting areas) also aligns with the flow and waiting time data documented in this study. For example, the lengthy appointment booking time resulted in over-crowding of the common space and undue stress on patients as they waited to book their appointments. Process improvements have now been implemented to facilitate appointment booking and decrease the time of that work step. Given the relatively low proportion of personnel and facility usage cost in the total costs, improving efficiencies in the patient flow are not likely to result in major cost savings to the system. However, improving efficiency is of the utmost importance in order to be well-equipped to handle the increasing number of patients going through the cancer trajectory, and is a process that has been implemented at other cancer facilities [20-22].

## Conclusions

In conclusion, by combining time and motion methodology with information from hospital databases we determined that in the context of increasingly expensive therapies, the relative contribution of personnel and O&M costs to the overall cost is significantly smaller than expected. The methodology in this study is a “bottom-up” approach which only accounts for salaries attributed to our study cases. However, a significant portion of work time is typically spent on tasks not attributable to specific cases and which we have not fully accounted for (with the exception of O&M costs). We have also excluded physician salary costs in this analysis because they are not hospital-based expenses in Quebec. In contrast, when analyzed for an entire health care system, salaries typically comprise a significant portion of the costs, and in general hospital care is provided with medications that are not as expensive as those for cancer, with the number of person-hours considerably higher. Thus in other countries with different health care structures, personnel costs for physician and treatment visits for cancer care may account for a greater proportion of the costs.

This study has also provided quantitative information about patient and work flow which when used in conjunction with what has been observed in the clinic and what patients have said, has helped guide the implementation of process changes to increase efficiency. With the emerging paradigm of 'personalized' medicine in which molecular diagnostic testing increasingly guides treatment selection, at times also requiring tumour biopsies for testing, there will be new components in the cancer treatment teams. Molecular pathologists, interventional radiologists and their respective technical support may have to be added to the already complex flow chart. Our approach can be used as a template to examine the impact of these changes, as health care managers decide on allocating these resources.

## Abbreviations

O&M: Operation and Maintenance; RA: Research Assistant; FOLFOX: Folinic Acid (Leucovorin); Fluorouracil; Oxaliplatin; XELOX: Capecitabine; Oxaliplatin.

## Competing interests

During this study, Pierre Emmanuel Paradis and Natalia Mishagina were full time employees of Groupe d’analyse ltée, which had a consulting agreement with Gerald Batist to design the study and perform the data analysis.

## Authors’ contributions

GS designed the study protocol, supervised the data collection, contributed to the analysis of the data and wrote the manuscript. PEP designed the study protocol, analyzed the data and edited the manuscript. MP collected the data, participated in revisions to the study protocol and reviewed the manuscript. NM analyzed the data and reviewed the manuscript. M-PG and DL participated in the design of the study protocol and data collection and reviewed the manuscript. GB participated in the design of the study protocol, provided patients for the study and wrote the manuscript. All authors read and approved the final manuscript.

## Authors’ information

GS holds a doctoral degree with a research background in molecular biology and is the Program Coordinator for the McGill Department of Oncology. PEP is an economist and finance specialist. NM is an economist with a PhD in economics. M-PG is a pharmacist and the oncology pharmacy coordinator. DL is the nurse manager for the oncology clinic. GB is a medical oncologist and the Director of the Segal Cancer Centre as well as a Professor in the McGill Department of Oncology.

## Pre-publication history

The pre-publication history for this paper can be accessed here:

http://www.biomedcentral.com/1472-6963/12/370/prepub
